# Ultrasound Assessment of the Tibial Nerve at the Retromalleolar Level: Influence of Anthropometric Characteristics and Clinical Implications

**DOI:** 10.3390/clinpract15120227

**Published:** 2025-12-03

**Authors:** María Benimeli-Fenollar, Cecili Macián-Romero, Lucía Carbonell-José, María José Chiva-Miralles, José Maria Montiel-Company, José Manuel Almerich-Silla, Rosa Cibrian, Vicent Tomás-Martínez

**Affiliations:** 1 Department of Nursing, University of Valencia, c/Jaume Roig s/n, 46010 Valencia, Spain; cecili.macian@uv.es (C.M.-R.); lucia.carbonell@uv.es (L.C.-J.); maria.jose.chiva@uv.es (M.J.C.-M.); 2 Department of Stomatology, University of Valencia, c/Gascó Oliag, 1, 46010 Valencia, Spain; jose.maria.montiel@uv.es (J.M.M.-C.); jose.m.almerich@uv.es (J.M.A.-S.); 3 Department of Physiology, University of Valencia, c/Blasco Ibánez, 15, 46010 Valencia, Spain; rosa.m.cibrian@uv.es; 4 Independent Researcher, 46010 Valencia, Spain; vicenttomasmartinez@gmail.com

**Keywords:** tibial nerve, ultrasound imaging, retromalleolar region, posterior tibial artery, regional anesthesia, ankle block, anthropometry, anatomical variability, tarsal tunnel syndrome, neuropathy

## Abstract

**Background:** Clinical procedures involving the tibial nerve (TN) are complex procedures due to its deep anatomical position and the variability of its course in the retromalleolar region. Few studies have described the ultrasound characteristics of the TN in vivo. This study aims to describe the ultrasound position of the TN and its relationship with the posterior tibial artery (PTA) at the retromalleolar level, evaluating the influence of sex, weight, height, and body mass index (BMI). **Methods:** A cross-sectional ultrasound study was performed on 100 volunteers. Anthropometric variables were recorded. Ultrasound measurements included the TN perimeter, distance from the medial malleolus to the TN center, depth, and spatial relationship with the PTA. Statistical analyses included Student’s *t*-test, ANOVA, Chi-square test, and Pearson’s correlation coefficient, with a significance level of *p* < 0.05. **Results:** The mean distance from the TN to the medial malleolus was 2.17 cm, and its mean depth was 0.91 cm. The most common anatomical pattern was Type I (TN posterior to the PTA) (60%). Sex influenced TN position, with men showing greater distances from the medial malleolus to the TN center (2.42 vs. 1.99 cm) and women showing greater depth from the skin surface to the upper edge of the tibial nerve perimeter (0.94 vs. 0.86 cm). Weight (*p* = 0.004), height (*p* < 0.001), and ankle circumference (*p* = 0.006) correlated significantly with TN location, whereas BMI did not (*p* = 0.253). **Conclusion:** These findings provide clinically relevant reference data that may improve the precision and safety of different tibial nerve procedures.

## 1. Introduction

The clinical relevance of accurately locating the tibial nerve (TN) lies in the fact that it must be anesthetized in many feet surgical procedures. Because of its deep anatomical position and close relationship with the posterior tibial artery (PTA), performing tibial nerve blocks can be technically challenging [1,2,3,4,5]. Accurate knowledge of its anatomical variability is essential to enhance the safety and success rate of regional anesthesia. In addition, awareness of the subject’s anthropometric characteristics may also contribute to optimizing other clinical procedures involving the tibial nerve, including infiltrations for tarsal tunnel syndrome and diagnostic evaluations of tibial nerve neuropathy.

Previous anatomical studies have been conducted mainly on cadaveric specimens [6,7,8,9,10,11,12]. Although these investigations provide valuable structural information, the manipulation inherent to cadaveric dissection can alter the natural positions of the tibial nerve and the posterior tibial artery, thereby reduce the accuracy of anatomical relationships when extrapolate to living subjects. High-resolution ultrasound has become a well-established and effective tool for real-time visualization of peripheral nerves enabling more precise localization for both diagnostic and anesthetic purposes.

Understanding how anthropometric factors such as sex, height, weight, and ankle circumference affect the TN’s position, may help clinicians anticipate variations and plan safer puncture sites. However, no studies have analyzed these correlations using ultrasound imaging in a living population.

The present study aims to describe the ultrasound characteristics of the tibial nerve at the retromalleolar level and to evaluate the influence of anthropometric variables on its anatomical position. By establishing reliable reference coordinates, we seek to provide clinically relevant data that improve the accuracy and safety of tibial nerve invasive procedures in daily practice. The hypothesis of the study was that the subject’s sex, weight, height, and BMI influence the position of the nerve and its relationship with the posterior tibial artery.

## 2. Materials and Methods

### 2.1. Study Design and Participants

A descriptive, cross-sectional ultrasound study was conducted at the University the Valencia to evaluate the anatomical position and morphometric characteristics of the tibial nerve at the retromalleolar level in 100 healthy adult volunteers (58 women and 42 men), aged between 18 and 75 years. Participants were recruited through public announcements within the university community.

Inclusion criteria were absence of lower limb neuropathies, previous trauma or surgery in the ankle region, and no history of systemic conditions affecting peripheral nerves (e.g., diabetes mellitus, polyneuropathy). Exclusion criteria included: presence of edema, varicose veins, or any anatomical deformity that could alter ultrasound visibility of the tibial nerve (TN) or the posterior tibial artery (PTA).

The study was conducted following the ethical standards of the Declaration of Helsinki and was approved by the Valencia University Ethics Committee (approval code: H1477566491165). All participants signed written informed consent prior to data collection.

### 2.2. Calibration

The calibration phase was conducted under the supervision of a clinician with extensive experience in musculoskeletal ultrasound, with the aim of standardizing the measurement protocol. During this phase, each observer independently identified the tibial nerve in a transverse ultrasound image at the medial aspect of the ankle and measured the different study variables in a total of 15 subjects. The participants included in the calibration phase were not included in the final analysis.

An intraclass correlation coefficient was obtained for the variables: distance from the bony reference point to the center of the tibial nerve, and depth of the tibial nerve: 0.956 (95% CI: 0.891–0.982) and 0.981 (95% CI: 0.952–0.993), respectively.

The variable tibial nerve perimeter yielded an intraclass correlation coefficient of 0.547 (95% CI: 0.142–0.793). Diagnostic agreement for the type of anatomical relationship between the neurovascular structures, between both examiners, was 100%, with a Kappa value of 1, indicating very good agreement according to the Landis and Koch scale.

### 2.3. Ultrasound Protocol

Ultrasound examinations were performed by an experienced sonographer using a high-resolution linear transducer (4–12 MHz) (Vinno 5^®^) (Vinno Technology Co., Suzhou, China). Participants were placed in a supine position with the knee flexed, the hip externally rotated and the ankle joint in a neutral position.

**Figure 1:**
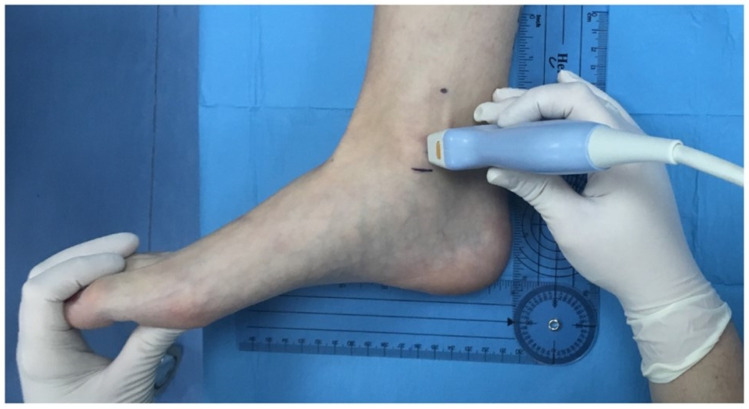
Localization and marking of the bony reference points for the retromalleolar study of the tibial nerve. Position of the ultrasound probe over the most prominent point of the medial malleolus, with the ankle joint in a neutral position.

**Figure 2:**
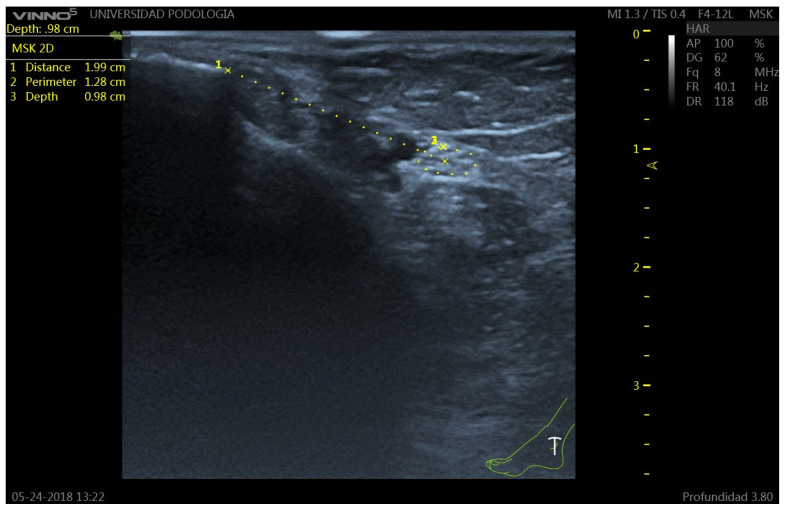
Ultrasound image of the tibial nerve (TN) at the retromalleolar level. The TN appears as a hyperechoic structure with internal hypoechoic fascicles (honeycomb pattern), posterior to the posterior tibial artery (PTA). 1: TN-malleolus distance; 2: TN perimeter; 3: Perpendicular distance from the skin surface to the upper edge of the tibial nerve perimeter.

The probe was placed transversely at the level of the medial malleolus to identify the tibial nerve (TN) and the posterior tibial artery (PTA) (Figure 1). The TN was recognized as a hyperechoic oval structure with a fascicular “honeycomb” pattern, located posterior or lateral to the PTA (Figure 2).

**Figure 3:**
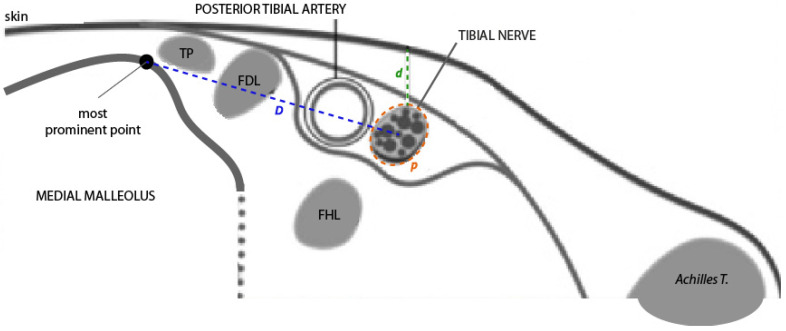
Diagram of the ultrasound view of the neurovascular bundle in a transverse section at the retromalleolar level. From right to left, note the following structures: the posterior tibial tendon (TP), the flexor digitorum longus tendon (FDL), the posterior tibial artery, and the tibial nerve. The flexor hallucis longus tendon (FHL) is in a deeper plane. Measurement of positional variables: *D* = horizontal distance at the skin surface from the bony reference point to the center of the tibial nerve; *p* = perimeter of the tibial nerve; *d* = vertical distance (depth) from the skin surface to the upper edge of the tibial nerve perimeter. Source: authors owns elaboration.

Measurements were obtained at the level of the most prominent point of the medial malleolus. The following parameters were recorded (Figure 3):

1. **Distance (D):** from the most prominent point of the medial malleolus to the center of the TN.
2. **Depth (d):** perpendicular distance from the skin surface to the upper edge of the tibial nerve perimeter.
3. **Perimeter (P):** measured along the outer contour of the TN.
4. **TN–PTA relationship:** classified according to the relative position of both structures.

Each measurement was performed three times, and the mean value was used for statistical analysis.

#### 2.3.1. Classification of Nerve–Artery Relationship

**Figure 4:**
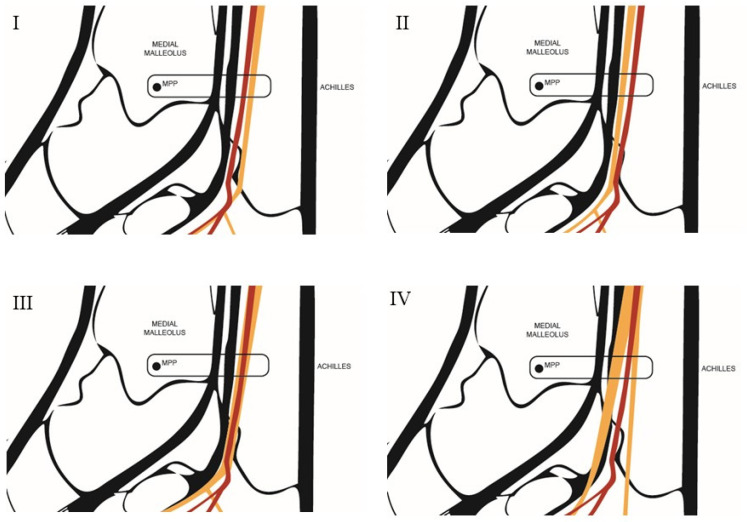
Diagram of the anatomical relationship types between the tibial nerve and the posterior tibial artery. Type **I**: The tibial nerve is located posterior to the posterior tibial artery. Type **II**: The tibial nerve is located anterior to the posterior tibial artery. Type **III**: The tibial nerve is in a lateral position relative to the posterior tibial artery. Type **IV**: A bifurcation of the tibial nerve is observed. MPP = Most prominent point. Source: Authors owns elaboration.

The classification of the spatial relationship between the tibial nerve and the posterior tibial artery used in this study follows the anatomical system proposed by Kim et al. [6]. Four types of relationships were established between the TN and the PTA (Figure 4):

- Type I: When, after outlining the perimeter of the tibial nerve, the nerve is located posterior to the posterior tibial artery.
- Type II: When, after outlining the perimeter of the tibial nerve, the nerve is located anterior to the posterior tibial artery.
- Type III: When, after outlining the perimeter of the tibial nerve, the nerve trunk is positioned beneath the vascular bundle, in a lateral position relative to the posterior tibial artery.
- Type IV: When more than one trunk corresponding to the tibial nerve is observed.

The distribution of these patterns was recorded for the total sample and by sex.

#### 2.3.2. Anthropometric Variables

Anthropometric data included: Age (years), Sex (male/female), Weight (kg), Height (cm), Body mass index (BMI, kg/m^2^) and Ankle circumference (cm), measured at the level of the medial malleolus using a flexible tape with the participant standing.

All measurements were collected by the same investigator to minimize inter-observer variability.

### 2.4. Statistical Analysis

The data obtained were entered into an Excel spreadsheet and analyzed using IBM SPSS Statistics, version 22, IBM Corp., Armonk, NY, USA. Means were calculated for quantitative variables and proportions for categorical variables, along with their corresponding 95% confidence intervals (CI).

Comparisons of means were performed using Student’s *t*-test and one-way ANOVA, with Bonferroni correction as the post hoc test. The normality of distributions was assumed according to the central limit theorem for samples larger than *n* = 30.

For comparisons of proportions, the Chi-square test was applied. In addition, the existence of a linear relationship between quantitative variables was assessed using Pearson’s correlation coefficient.

A *p*-value of <0.05 was considered statistically significant.

## 3. Results

### 3.1. Participant Characteristics

The study included 100 volunteers (58 women and 42 men). The mean age of the total sample was 27.5 (19–65) years. Table 1 shows the main anthropometric characteristics of the participants.

### 3.2. Ultrasound Measurements of the Tibial Nerve

Tibial nerve was located at an average distance of 2.17 cm (range, 1.03–3.28 cm) from the most prominent point of the medial malleolus, and at an average depth of 0.91 cm (range, 0.46–1.39 cm) from the skin surface (Table 2).

### 3.3. Anatomical Relationship Between the Tibial Nerve and Posterior Tibial Artery

**Figure 5:**
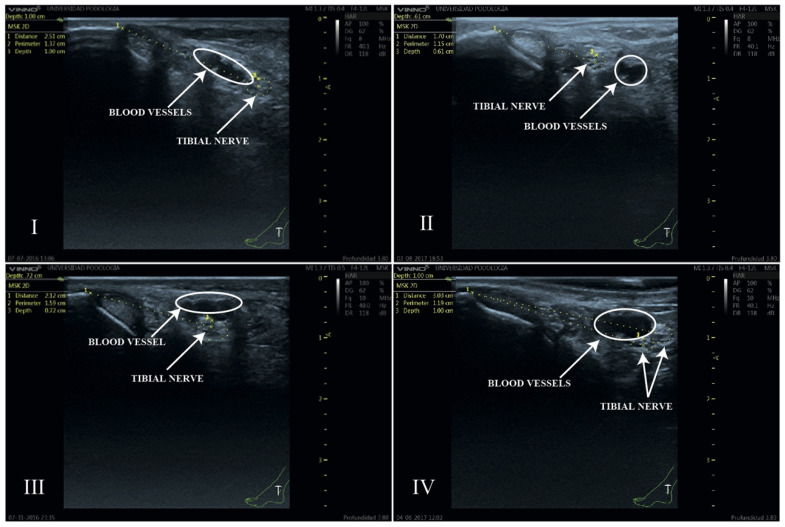
Ultrasound image of the four anatomical relationships between the TN and the PTA. Type **I**: TN posterior to PTA; Type **II**: TN anterior to PTA; Type **III**: TN lateral to PTA; Type **IV**: bifurcated TN. The white circles indicate the anatomical position of the blood vessels. The location of the tibial nerve has been defined by the yellow dashed circle of its perimeter.

**Figure 6:**
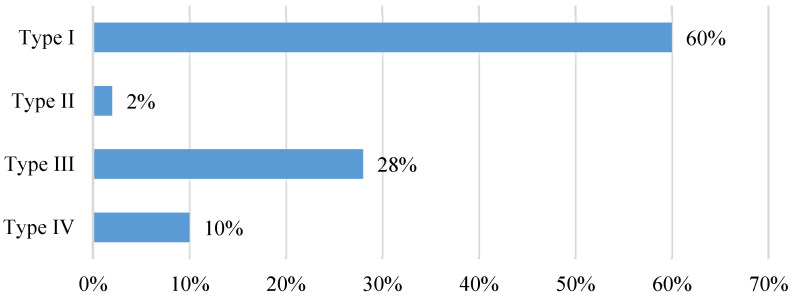
Anatomical relationship between the tibial nerve and the posterior tibial artery observed in the ultrasound study at the retromalleolar level. Type of anatomical relationship: Type I: TN posterior to PTA; Type II: TN anterior to PTA; Type III: TN lateral to PTA; Type IV: bifurcated TN).

Four anatomical patterns were identified (Figure 5). The most frequently observed position of the tibial nerve within the neurovascular bundle (60%) was the one in which the nerve was located posterior to the artery (Type I). A bifurcation of the tibial nerve (Type IV) at the retromalleolar level was observed on up to ten occasions (10%) (Figure 6).

### 3.4. Analysis of Tibial Nerve Position by Sex and BMI Category

**Figure 7:**
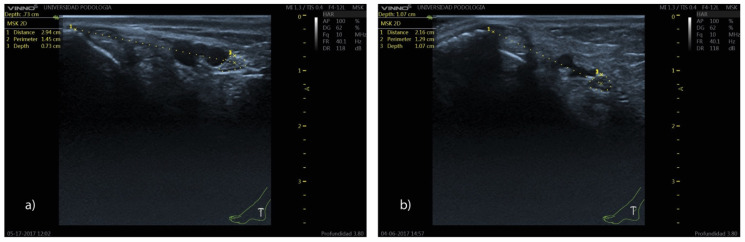
Ultrasound image showing the position of the tibial nerve at the retromalleolar level in men (**a**) and women (**b**). Note that the distance from the bony reference point to the center of the tibial nerve is greater in men (**a**) (2.94 cm vs. 2.16 cm), whereas the depth relative to the skin surface is greater in women (**b**) (0.73 vs. 1.07 cm).

The analysis of tibial nerve position variables by sex and BMI category is summarized in Table 3. Men showed a significantly greater distance from the most prominent point of the medial malleolus to the center of TN (*p* < 0.01), while women had slightly greater nerve depth (*p* < 0.05) (Figure 7). No significant differences were found in the position of the tibial nerve (TN-malleolus distance and TN depth) across the different BMI categories.

### 3.5. Analysis of Anatomical Relationship Between the Tibial Nerve and Posterior Tibial Artery by Sex and BMI Category

The results of the analysis of the anatomical relationship between the tibial nerve and the posterior tibial artery by sex and BMI category are summarized in Table 4. 

With a *p*-value < 0.001 obtained using the Chi-square test, statistically significant differences were found in the position of the tibial nerve within the neurovascular bundle between men and women. A greater predominance of Type I positioning was observed in women, whereas Types III and IV were more common in men. In contrast, no significant differences were found in the position of the tibial nerve across the different BMI categories (*p*-value = 0.602).

### 3.6. Analysis of Anatomical Relationship Between the Tibial Nerve and Posterior Tibial Artery by Weight, Height, BMI and Ankle Circumference

The results of the analysis of the anatomical relationship between the tibial nerve and the posterior tibial artery according to weight, height, BMI, and malleolar circumference are summarized in Table 5.

The ANOVA test revealed significant differences in the position of the tibial nerve within the neurovascular bundle among subjects with varying weight (*p*-value = 0.004), height (*p*-value = 0.000), and malleolar circumference (*p*-value = 0.006).

The Bonferroni post hoc test showed that these differences were mainly found between Type I and Type III positions for the variables weight (*p*-value = 0.004) and height (*p*-value = 0.001), and between Type I and Type IV positions for the variable malleolar circumference (*p*-value = 0.008).

### 3.7. Correlation Analysis

Table 6 presents the results obtained from Pearson correlation analyses among the quantitative variables of the study.

A positive linear correlation was observed between the distance from the most prominent point of the medial malleolus to the center of the tibial nerve and the variables weight (Pearson’s *r* = 0.340), height (Pearson’s *r* = 0.409), and malleolar circumference (Pearson’s *r* = 0.303). 

A positive linear correlation was also found between malleolar circumference and age (Pearson’s *r* = 0.245) and BMI (Pearson’s *r* = 0.496), and therefore, indirectly, with the subject’s weight and height.

These findings indicate that taller and heavier individuals tend to have the TN located farther from the medial malleolus but at similar depths.

To facilitate interpretation of the main findings, Table 7 summarizes the results in which statistically significant differences were identified (*p* < 0.05). These include sex-related differences in tibial nerve position, as well as the influence of weight, height, and ankle circumference on the anatomical relationship between the tibial nerve and the posterior tibial artery. This focused summary highlights the variables that demonstrated a measurable effect on tibial nerve location and neurovascular configuration at the retromalleolar level.

## 4. Discussion

The initial hypothesis of the present study was that the subject’s sex, weight, height, and BMI could influence the position of the nerve and its relationship with the posterior tibial artery.

The results obtained in this study revealed that, at the retromalleolar level, the subject’s sex affects both the distance from the bony reference point to the center of the tibial nerve and the depth at which the tibial nerve is located. Furthermore, sex, weight, height, and malleolar circumference were found to influence the position of the tibial nerve within the neurovascular bundle and its relationship with the posterior tibial artery at the retromalleolar level.

The anatomical reference line used to perform the various measurements was the imaginary horizontal line passing through the most prominent point of the medial malleolus. This anatomical reference line served as the zero point from which all measurements were taken. The selection of this anatomical reference point was based, first, on its easy identification through palpation, and second, on the fact that placing the transducer over the most prominent point of the medial malleolus provided a highly specific and easily recognizable ultrasound image, ensuring correct probe positioning.

All measurements were conducted with the foot positioned at a 90° angle to the tibia to standardize the obtained results and minimize potential calculation errors [6,9,12,13,14,15]. As in the most recent anatomical studies [14,15], distance measurements were taken using a digital caliper rather than a conventional ruler to ensure maximum precision in the results. In all cases, plantar flexion of the hallux was performed to avoid confusing the ultrasound image of the flexor hallucis longus tendon with that of the tibial nerve structure [16]. 

As in other studies [6,9,17], two anatomical axes were established to perform the various measurements: an X-axis for transverse coordinates and a Y-axis for vertical coordinates. The lack of standardized guidelines defining how to classify the anatomical relationship between neurovascular structures at the ankle level has made it difficult to compare both methodology and results with those of other studies. To date, the only classification described for analyzing the anatomical relationship between the tibial nerve and the posterior tibial artery is the one proposed by Kim et al. [6]. This classification is based on the position of the posterior tibial artery relative to the tibial nerve and describes four types of relationships depending on whether the artery is located medial, anterior, or lateral to the tibial nerve, or positioned between the medial and lateral plantar nerves. In the present study, the tibial nerve was used as the anatomical reference structure.

The most frequently observed relationship in the study by Kim et al. [6] carried out in cadaveric specimens, was one in which the posterior tibial artery is located medial to the tibial nerve (55.6%). In our study, the tibial nerve was located lateral to the artery (Type III) in only 5% of cases at the retromalleolar level. In contrast, the relationship type with the highest prevalence in the present study was the one in which the nerve was positioned posterior to the artery (Type I) (85%). Likewise, the number of cases in which the tibial nerve appeared bifurcated was considerably lower in our study (5%) compared with the 17.8% reported in the study by Kim et al. Both studies corroborate that the posterior tibial artery tends to adopt a more anterior position relative to the tibial nerve as it progresses distally.

These results show that in vivo visualization of the neurovascular bundle is of great importance when describing the exact position of a nerve structure, as it can provide complementary information to that obtained from conventional anatomical studies on cadavers, or even differ from them in certain key aspects.

Studies of the tibial nerve conducted on anatomical specimens have focused primarily on describing the bifurcation pattern of the tibial nerve at the level of the tarsal tunnel, the number of terminal branches, and the origin of the medial and inferior calcaneal branches [7,8,9,10,11,12,17,18]. These investigations have provided relevant information on the anatomical variability of the nerve along its distal course, but they have not assessed its spatial location in relation to external anatomical landmarks.

To the best of our knowledge, no previous studies have determined the distance or depth of the tibial nerve in relation to a fixed bony reference point at the retromalleolar level, either in cadaveric specimens or in vivo subjects. This lack of comparative data limits the possibility of directly contrasting our findings with those from other investigations and underscores the relevance of providing reference values obtained from a large sample of healthy participants using ultrasound.

In fact, one of the main findings of this study is that the results of the analysis of nerve position variables (distance from the bony reference point, depth, and position of the nerve within the neurovascular bundle) vary according to the subject’s anthropometric characteristics.

The average distance from the bony reference point to the center of the tibial nerve is greater in men, the nerve is located between 2.28 and 2.54 cm from the most prominent point of the medial malleolus and in female subjects, between 1.91 and 2.08 cm. The greater distance between the tibial nerve and the bony reference point observed in men may be explained by sex-related anatomical differences. In general, men exhibit greater calf muscle thickness, particularly in the gastrocnemius and soleus muscles, as well as a larger volume of connective and fascial tissue in the posterior compartment of the leg. This increased soft-tissue thickness between the medial malleolus and the tibial nerve can enlarge the measured linear distance [19,20].

In our analysis, the tibial nerve is located at a greater depth in women. However, it still follows a relatively superficial course at the retromalleolar level, with a mean depth of 0.91 cm. Therefore, the tibial nerve cannot be characterized as a deep structure at this anatomical location. Knowing the mean depth in woman (0.94 cm) and in men (0.86 cm) at which the tibial nerve is located can be extremely useful as a reference parameter to help prevent nerve lacerations during invasive procedures. The greater depth of the tibial nerve observed in females may be explained by known anatomical and compositional differences between men and women in the ankle and lower-leg region. Although men typically exhibit greater calf muscle mass, women generally present a relatively thicker layer of subcutaneous adipose tissue, particularly in the lower limbs. This increased adipose layer can enlarge the distance between the skin surface and deeper structures, including the tibial nerve. Moreover, anthropometric studies have shown that women tend to have greater thickness of superficial soft tissues, even when deep muscle mass is lower, which aligns with the slightly greater depth observed in our analysis [21,22].

A greater predominance of the tibial nerve in a position posterior to the posterior tibial artery (PTA) was observed in women, whereas in men, the tibial nerve more frequently occupied a lateral position relative to the PTA or appeared bifurcated. It was also observed that subjects weighing more than 71 kg had a higher likelihood of the tibial nerve being in a lateral position relative to the PTA at the retromalleolar level. Similarly, a height greater than 1.72 m increased the probability of finding the tibial nerve in a lateral position within the neurovascular bundle or bifurcated at the level of the most prominent point of the medial malleolus. This finding may be clinically relevant, as it can help position the needle as close as possible to the nerve trunk. These observations may tentatively be explained by the fact that, in men, greater muscle volume could displace the neurovascular bundle laterally or alter its course within the tarsal tunnel. Likewise, a longer distal limb segment in taller individuals might facilitate the bifurcation of the tibial nerve at a more proximal point, which would be consistent with the higher prevalence of bifurcations observed in taller participants. However, these interpretations should be considered hypothetical, and future studies are needed to confirm whether these anatomical tendencies truly account for the patterns identified in our sample.

An example of the clinical applicability of these findings in TN anesthetics blocks is the study carried out also by Benimeli et al. [5]. The authors performed a total of 55 tibial nerve blocks in 55 subjects, including 35 women (63.6%) and 20 men (36.4%). Tibial nerve block was performed by landmark technique in all cases with the subject’s foot positioned at 90° relative to the tibia, using a Braun^®^ BL/L 23G 0.60 × 25 mm needle and a conventional 5 mL syringe. The anesthetic solution administered consisted of 3 mL of 2% mepivacaine without vasoconstrictor (Scandinibsa^®^, Laboratorios Inibsa, Barcelona, Spain. National Code: 603031.8). For male subjects, the needle was inserted between 2.28 and 2.54 cm from the most prominent point of the medial malleolus, directed toward the Achilles tendon; and for female subjects, between 1.91 and 2.08 cm. The needle angle was 90° for individuals shorter than 1.72 m, and 45° for those with a height equal to or greater than 1.72 m. The insertion depth was consistent across all subjects: 0.90 cm from the skin surface. The percentage of vascular puncture and peri/intraneural puncture was 10.9% and 9.1%, respectively. Subjects rated the pain perceived during the anesthetic process as low on the VAS, 2.16 points from 0 to 8. A success rate of 81.8% was recorded for the described retromalleolar technique, with 18.2% of partial blocks and no failed blocks. Although a 100% success rate was not achieved, the number of effective blocks was considerably higher than those reported for other conventional techniques, which are not based on high-volume tibial nerve blocks (22% and 66% success rates, respectively). In fact, the success rate achieved with the retromalleolar technique in Benimeli’s study [5] was higher than that reported by Redborg et al. for the ultrasound-guided approach (72%). This author also report that ultrasound-guided blockade requires additional time to perform, and that 44.4% of subjects required three or more needle redirections [16].

The results described above may be explained by the fact that, unlike previous studies based on cadaveric dissection of the neurovascular bundle [6,8,10,13,17], the present ultrasound study of the tibial nerve allowed for direct in vivo observation of the neurovascular bundle. Understanding the anatomical position of the tibial nerve at the retromalleolar level may assist clinicians in anticipating the usual location of the nerve during regional anesthesia procedures, particularly in cases where real-time ultrasound guidance is not fully achievable.

The present study provides descriptive anatomical reference values for the tibial nerve in the retromalleolar region, including its depth, position relative to the posterior tibial artery, and the frequency distribution of anatomical variants. These findings offer clinicians a contextual framework for understanding the usual anatomical configuration of the tibial nerve in this area. This is particularly valuable for anesthesiologists, neurologists, podiatrists and physiotherapists in improving the efficacy and safety procedures such as retromalleolar tibial nerve block for foot and ankle surgeries, ultrasound-guided steroid or anesthetic injections for tarsal tunnel syndrome and diagnostic assessment of TN entrapment or neuropathy. Although repeatability is one of the main limitations of ultrasound, particularly in musculoskeletal and peripheral nerve imaging, the use of a standardized protocol and objective, reproducible measurements help minimize such variability. Thanks to advances in diagnostic imaging and improvements in clinical skills, sonographers from different backgrounds can obtain consistent ultrasound measurements with minimal differences and low variability in outcomes [23].

However, these values should not be interpreted as fixed or prescriptive puncture coordinates. Rather, they represent population-based reference measurements that may support clinical reasoning when planning procedures involving the tibial nerve. Before performing any invasive procedure, it is essential to consider the patient’s anatomical and anthropometric characteristics and, whenever possible, to carry out a real-time ultrasound visualization of the relevant structures. Accordingly, our results are intended solely as descriptive anatomical parameters and do not replace the safety principles or decision-making processes intrinsic to ultrasound-guided techniques.

### Study Limitations

This study presents several limitations that should be acknowledged to properly interpret the findings. First, although the sample size was relatively large, its demographic composition, predominantly young and normal-weight adults, may not fully represent the broader population undergoing invasive procedures in the retromalleolar region. Consequently, the anatomical reference values reported here should be regarded as population-based parameters rather than categorical recommendations. However, we consider it relevant to note that numerous tibial nerve blocks are performed in young individuals without nerve pathology, particularly in minor surgical procedures such as the excision of skin lesions (plantar warts, nevi, inclusion corns) or in osteoarticular forefoot surgeries that also require tibial nerve anesthesia. In this group of patients, clinically comparable to our study sample, the anatomical reference values provided by this research may be especially useful to optimize the precision and safety of the procedure.

Individuals with neuropathies, local deformities, edema, or inflammatory conditions were excluded. Therefore, the results cannot be extrapolated to patients with pathological alterations such as tarsal tunnel syndrome, where nerve enlargement or changes in the neurovascular bundle may significantly modify anatomical relationships.

Although intra-observer reliability was assessed, the study did not evaluate inter-observer reproducibility. Future research including multiple independent examiners would help determine the generalizability of the measurement protocol.

Cross-sectional area (CSA) is the most used parameter in nerve ultrasonography as a morphological measure of the nerve. However, in our study carried out in healthy participants, we have used the perimeter and not this parameter to accurately determine the anatomical variable “tibial nerve depth”, defined as the distance from the skin surface to the upper border of the tibial nerve.

This study did not correlate anatomical findings with clinical or surgical outcomes. However, the anatomical reference data generated in this study have been used and validated in a previous clinical study conducted by the same authors, which provides additional, though external, value to the present work.

Finally, despite the strengths of high-resolution ultrasound, this technique remains operator dependent. Nonetheless, recent advances in musculoskeletal and nerve imaging have shown that, with standardized protocols and objective, repeatable measurement criteria, sonographers from different backgrounds can obtain highly consistent results with minimal variability.

Despite these limitations, the findings provide relevant and previously unavailable anatomical data on the position of the tibial nerve at the retromalleolar level, which may serve as a basis for future research and enhance the clinical understanding of this region.

## 5. Conclusions

The tibial nerve at the retromalleolar level demonstrates a consistent but variable relationship with the posterior tibial artery, predominantly posterior in position. Anthropometric factors, particularly sex, height, weight, and ankle circumference, significantly influence its location, whereas BMI does not.

These findings establish ultrasound-based anatomical reference data that can enhance both diagnostic accuracy and the clinical safety of procedures involving the tibial nerve. Future studies including larger and more diverse populations may help refine these reference parameters for broader clinical application.

## Figures and Tables

**Table 1:** Descriptive anthropometric characteristics of participants.

Variable	Mean (Range)	95% CI
Age (years)	27.5 (19–65)	[25.6–29.5]
Height (m)	1.68 (1.51–1.93)	[1.66–1.70]
Weight (Kg)	65.3 (40–105)	[62.7–67.9]
BMI (kg/m^2^)	22.8 (16.6–36.3)	[22.1–23.5]
Ankle circumference (cm)	24.9 (22–29)	[24.5–25.2]

**Table 2:** Results of the ultrasound measurements of the tibial nerve (TN) in the study sample.

Variable	Mean (Range)	95% CI
TN-malleolus distance (cm)	2.17 (1.03–3.28)	[2.09–2.25]
TN Depth (cm)	0.91 (0.46–1.39)	[0.86–0.95]
TN perimeter (mm)	1.35 (0.96–1.9)	[1.31–1.39]

**Table 3:** Analysis of the positional variables of the tibial nerve according to sex and the subject’s BMI category.

			*N*	Mean	95% CI	Statistical Test
TN-malleolus distance	Sex	Woman	58	1.99	[1.91–2.08]	Student *t* *p* value < 0.001 *
		Man	42	2.42	[2.28–2.54]	
	BMI category	1	6	2.19	[2.04–2.34]	ANOVA test *p* value = 0.276
		2	66	2.25	[2.14–2.35]	
		3	25	2.39	[2.26–2.52]	
		4	3	2.49	[2.06–2.91]	
TN depth	Sex	Woman	58	0.94	[0.88–1.00]	Student *t* *p* value = 0.047 *
		Man	42	0.86	[0.79–0.92]	
	BMI category	1	6	0.77	[0.54–0.99]	ANOVA test *p* value = 0.055
		2	66	0.89	[0.84–0.94]	
		3	25	0,95	[0.86–1.04]	
		4	3	1.15	[0.66–1.65]	

BMI category: 1 = Underweight; 2 = Normal weight; 3 = Overweight; 4 = Obesity. * Significant difference *p* < 0.05.

**Table 4:** Analysis of the anatomical relationship between the tibial nerve and the posterior tibial artery by sex and BMI category in the ultrasound study sample at the retromalleolar level.

	Type I *N* = 60		Type II *N* = 2		Type III *N* = 28		Type IV *N* = 10		Statistical Test
	*N* (%)	95% CI	*N* (%)	95% CI	*N* (%)	95% CI	*N* (%)	95% CI	Chi^2^ Test
Woman	44 (75.9)	[63.5–85.0]	1 (1.7)	[0.3–9.1]	12 (20.7)	[12.3–32.8]	1 (1.7)	[0.3–9.1]	*p* value < 0.001 *
Man	16 (38.1)	[24.9–53.2]	1 (2.4)	[0.4–12.3]	16 (38.1)	[24.9–53.2]	9 (21.4)	[11.7–35.9]	
Cat. 1	6 (100.0)	[60.9–100]	0 (0.0)	[0–39.0]	0 (0.0)	[0–39.0]	0 (0.0)	[0–39.0]	*p* value = 0.602
Cat. 2	39 (59.1)	[47.0–70.1]	2 (3.0)	[0.8–10.4]	17 (25.8)	[16.7–37.4]	8 (12.1)	[6.3–22.1]	
Cat. 3	13 (52.0)	[33.5–69.9]	0 (0.0)	[0–13.3]	10 (40.0)	[23.4–59.3]	2 (8.0)	[2.2–24.9]	
Cat. 4	2 (66.7)	[20.7–93.8]	0 (0.0)	[0–56.1]	1 (33.3)	[6.1–79.2]	0 (0.0)	[0–56.1]	

Type of anatomical relationship: Type I: the TN is located posterior to the PTA; Type II: the TN is located anterior to the PTA; Type III: the TN is positioned lateral to the PTA; Type IV: More than one nerve trunk corresponding to the TN is observed. BMI category: 1 = Underweight; 2 = Normal weight; 3 = Overweight; 4 = Obesity. *: Significant difference *p* < 0.05.

**Table 5:** Analysis of the anatomical relationship between the tibial nerve and the posterior tibial artery according to weight, height, BMI, and malleolar circumference in the ultrasound study sample at the retromalleolar level.

	Type I *N* = 60		Type II *N* = 2		Type III *N* = 28		Type IV *N* = 10		ANOVA Test	Bonferroni POST HOC
	Mean	95% CI	Mean	95% CI	Mean	95% CI	Mean	95% CI		
Weight	61.5	[58.4–64.7]	67.5	[35.7–92.3]	71.7	[66.4–77.1]	69.8	[62.5–77.1]	*p* value = 0.004 *	Position 1 vs. 3 *p* value = 0.004
Height	1.65	[1.63–1.67]	1.72	[1.21–2.22]	1.72	[1.69–1.76]	1.77	[1.72–1.82]	*p* value < 0.001 *	Position 1 vs. 3 *p* value = 0.001
BMI	22.4	[21.6–23.2]	22.8	[20.1–25.6]	23.9	[22.4–25.4]	22.3	[20.1–24.5]	*p* value = 0.253	-
Malleolar circumference	24.5	[24.0–24.9]	24.3	[21.1–27.4	25.2	[24.6–25.8]	26.4	[25.2–27.5]	*p* value = 0.006 *	Position 1 vs. 4 *p* value = 0.008

Type of anatomical relationship: type I: the TN is located posterior to the PTA; type II: the TN is located anterior to the PTA; type III: the TN is positioned lateral to the PTA; type IV: More than one nerve trunk corresponding to the TN is observed. *: Significant difference *p* < 0.05.

**Table 6:** Correlations among tibial nerve position, malleolar circumference, age, weight, height, and BMI in the ultrasound study sample at the retromalleolar level (*N* = 100).

		TN Perimeter	TN Depth	Malleolar Circumference	Age	BMI	Heigh	Weigh
TN-Malleolus distance	Pearson Correlation (two-tailed sig.)	0.007 0.945	−0.188 0.061	0.303 ** 0.002	0.076 0.453	0.159 0.155	0.409 ** 0.000	0.340 ** 0.001
TN perimeter	Pearson Correlation (two-tailed sig.)		0.128 0.205	0.019 0.851	0.027 0.788	0.202 * 0.044	−0.192 0.055	0.038 0.707
TN depth	Pearson Correlation (two-tailed sig.)			−0.006 0.955	−0.057 0.573	0.190 0.059	−0.097 0.339	0.080 0.430
Malleolar circumference	Pearson Correlation (two-tailed sig.)				0.245 * 0.014	0.496 ** 0.000	0.699 ** 0.000	0.746 ** 0.000
Age	Pearson Correlation (two-tailed sig.)					0.308 ** 0.002	−0.033 0.745	0.202 * 0.044
BMI	Pearson Correlation (two-tailed sig.)						0.195 0.052	0.828 ** 0.000
Height	Pearson Correlation (two-tailed sig.)							0.706 ** 0.000

TN = tibial nerve. *: *p* < 0.05; **: *p* < 0.01.

**Table 7:** Summary table with results in which statistically significant differences were identified (*p* < 0.05).

Variable	Comparison	Significant Result	*p*-Value
TN-malleolus distance	Sex (men vs. women)	Men showed a greater TN-malleolus distance	*p* = 0.000
TN depth	Sex (men vs. women)	Women showed a greater TN depth	*p* = 0.047
TN-PTA relationship	Sex	Differences in distribution of Types I–IV (women predominantly Type I; men more Type III and IV)	*p* = 0.000
TN-PTA relationship	Weight categories	Significant differences mainly between Type I and Type III	*p* = 0.004
TN-PTA relationship	Height categories	Significant differences mainly between Type I and Type III	*p* = 0.000
TN-PTA relationship	Ankle circumference categories	Significant differences mainly between Type I and Type IV	*p* = 0.006
Correlation (Pearson)	TN-malleolus distance and weight	Positive correlation	*p* = 0.001
Correlation (Pearson)	TN-malleolus distance and height	Positive correlation	*p* = 0.000
Correlation (Pearson)	TN-malleolus distance and ankle circumference	Positive correlation	*p* = 0.002

## Data Availability

The data generated and analyzed during the current study are not publicly available due to privacy and ethical restrictions but are available from the corresponding author on reasonable request.
